# Sleep and retrieval practice both strengthen and distort story recollection

**DOI:** 10.1093/sleepadvances/zpae083

**Published:** 2024-11-16

**Authors:** Dan Denis, Carissa DiPietro, R Nathan Spreng, Daniel L Schacter, Robert Stickgold, Jessica D Payne

**Affiliations:** Department of Psychology, University of York, York, UK; Department of Psychology, University of Notre Dame, Notre Dame, IN, USA; Montreal Neurological Institute, McGill University, Montreal, QC, Canada; Department of Psychology, Harvard University, Cambridge, MA, USA; Department of Psychiatry, Beth Israel Deaconess Medical Center, Boston, MA, USA; Department of Psychiatry, Harvard Medical School, Boston, MA, USA; Department of Psychology, University of Notre Dame, Notre Dame, IN, USA

**Keywords:** sleep, retrieval practice, memory, testing, consolidation

## Abstract

Over time, memories lose episodic detail and become distorted, a process with serious ramifications for eyewitness identification. What are the processes contributing to such transformations over time? We investigated the roles of post-learning sleep and retrieval practice in memory accuracy and distortion, using a naturalistic story recollection task. Undergraduate students listened to a recording of the “War of the Ghosts,” a Native American folktale, and were assigned to either a sleep or wake delay group, and either a retrieval practice or listen-only study condition. We found higher accuracy after sleep compared to wake in the listen-only condition, but not in the retrieval practice condition. This effect was driven by participants in the wake, retrieval practice condition showing superior memory compared to the wake, listen-only condition. A similar pattern was found for memory distortion, with both sleep and retrieval practice being associated with more inferences of nonpresented, but story-related information, compared to the wake, listen-only condition. These findings suggest both sleep and retrieval practice contribute to narrative memory stabilization and distortion.

**Statement of Significance**

A critical question for our understanding of human memory is how memories are transformed over time. Our long-term memories tend to lack episodic detail, with the “gist” of the episode being preserved. We also integrate related memories, allowing us to generalize new information based on our past experiences. How does this evolution of memory occur? Using the classic “War of the Ghosts” story, we found that overnight sleep (compared to a day awake) both strengthens memory, while also increasing the number of inferences made about plausible, but nonpresented information. Strikingly, the same effects were achieved through post-learning retrieval practice. In conclusion, our results suggest two different pathways (post-learning sleep and retrieval practice) that promote long-term memory transformation.

Human memories are inevitably transformed and distorted over time, causing us to remember past events quite differently than the true original experience. Episodic detail is lost, with the “gist” of the narrative being preserved [1, 2]. Distortions of memory also appear, with memory for different but overlapping events combined, or forgotten details being replaced or fabricated in order to fit a comprehensive narrative in tune with our cultural schemas [3–6]. Such transformative processes confer a flexibility and generalizability to memory that may at times be more advantageous than a veridical reinstatement of the original experience. However, this flexibility comes at the cost of memory accuracy, which has important real-world implications when considering the reliability of eyewitness testimony [5, 7]. As such, understanding when and how these transformations of memory occur remains a central question in the cognitive psychology of human memory.

In this *Festschrift*, we honor and celebrate Dr. Robert Stickgold’s major contributions to the fields of sleep and circadian science. Of greatest relevance to the present article is Dr. Stickgold’s work in the domain of sleep-related memory consolidation. Going beyond the classic definition of memory consolidation that refers simply to the process of memories becoming more resistant to interference, Dr. Stickgold argued for an expansion of the term consolidation to encompass a much broader set of processes, particularly pertaining to consolidation during sleep [8–11]. Coining the phrase *memory evolution* [10], empirical work from Dr. Stickgold’s laboratory has shown how sleep actively improves [12–14] and selectively enhances the most relevant aspects of memory [15]. Work from Dr. Stickgold’s lab has also shown that sleep-dependent memory evolution fosters the integration of new memory traces into preexisting schemas [16–18], as well as facilitating the generation of new insights and inferences of previously learned information [19, 20]. This body of work has gone on to be replicated and extended by multiple independent labs around the world [21–24].

In the present article, we continue to explore Dr. Stickgold’s idea of sleep-dependent memory evolution, through the examination of the learning and consolidation of an unfamiliar story. For this, we turned to another eminent scholar of memory, Dr. Frederik Bartlett and his famous “War of the Ghosts” experiments [3]. The War of the Ghosts is a Native American folktale written in a disjointed, non-Western style unfamiliar to most college students [3]. In his original experiments, Bartlett had participants read the story twice before testing their memory for the story over varying time delays. He found that over time, not only did forgetting occur, but memory also became distorted and infused with misinformation. Such distortions in recall became magnified over time and with repeated recall attempts, as participants held onto the schema or gist while replacing story details to keep a complete narrative. The importations could be quite dramatic, especially if the delay between recall attempts was long [3]. Since Bartlett’s initial study, his results have been replicated under stricter experimental conditions [25].

It is striking that many of the memory transformations observed by Dr. Bartlett map onto the types of memory evolution shown by Dr. Stickgold to occur during sleep, particularly the generalization of new information (the War of the Ghosts story) into something that made sense based on existing schemas (growing up in a Western culture more than 100 years after War of the Ghosts was written). Thus, guided by Dr. Stickgold’s ideas regarding memory evolution during sleep [10, 26, 27], we set out to investigate whether the time-dependent changes shown in the recall of the War of the Ghosts are heightened across a night of sleep compared to a day spent awake.

An important debate in the sleep and memory literature is what the exact boundary conditions are that allow for a sleep benefit to occur [28]. In other words, which aspects of memory consolidation are truly sleep-*dependent*. Numerous studies have demonstrated that simply attempting to recall a memory shortly after the original event can bolster memory strength, a phenomenon known as the testing effect [29–31]. Several recent studies have suggested memory consolidation processes once thought of as being dependent on sleep can also be achieved through post-learning retrieval practice. For example, using a paired associates task, researchers found that when participants engaged in retrieval practice, this eliminates the typical benefit of sleep compared to wake [32, 33]. Similar results have also been observed for recall of spatial locations [34].

While these studies suggest that time-dependent memory strengthening is not uniquely dependent on sleep, prior work has not addressed whether other transformative aspects of memory evolution, such as memory integration and inference generation, can also be achieved through retrieval practice. Although evidence of retrieval-induced distortion of memory has been observed [35, 36], the degree and exact nature of these distortions relative to overnight sleep have yet to be examined. The War of the Ghosts story has several advantages as a tool for studying memory evolution, and for examining which aspects of evolution may (or may not) be dependent on sleep. Story recall represents the kind of recollection that people engage in naturalistically, in which memory for the “gist” of an event often replaces veridical recall. It enables us to study not only accurate retrieval of facts, but also the distortions and importations of new information that result from schema-based representations. As such, we had two main research aims:

1. How does sleep and retrieval practice impact memory accuracy? This was tested through examining the number of story elements correctly recalled, as well as the number of intrusions of nonstory elements (i.e. false memories).
2. For inaccurate memories, does sleep and/or retrieval practice primarily contribute to a simple loss of episodic detail, or a distortion of the original story? For memory distortions, we categorized distortions into four separate types, allowing us to carefully examine where exactly any sleep-*dependent* memory evolution may occur.

## Methods

### Participants

A total of 114 Harvard University undergraduate students (*M*_age_ = 20.1 years old, range 18–30 years, 62 females) participated in the study and were compensated for their time with either course credit or cash. Because the research design and effects of interest were novel, it was not possible to estimate effect size from previous studies to perform an a priori power analysis. However, we aimed to recruit 30 participants per group, which was considered achievable given time and financial constraints, and was consistent with many sleep and memory studies [15, 37–40]. The participants were native English speakers with normal or corrected-to-normal vision and free from any psychiatric disorders, sleep, drug, or alcohol problems. Participants were not taking any medications affecting the central nervous system. Informed consent was obtained for all participants, and the study was approved by the Harvard University and Beth Israel Deaconess Medical Center Internal Review Boards. The research was performed in accordance with the Declaration of Helsinki.

### Materials

A recorded version of the Native American folktale “The War of the Ghosts” served as the to-be-remembered material [3] (Supplementary Table S1). All participants completed a three-night sleep log and the Stanford Sleepiness Scale (SSS; a one-item questionnaire used to assess participant’s alertness in the moment [41]) at the start of each session (Supplementary Table S2). None of the included participants reported prior experience with the story.

**Figure 1.**
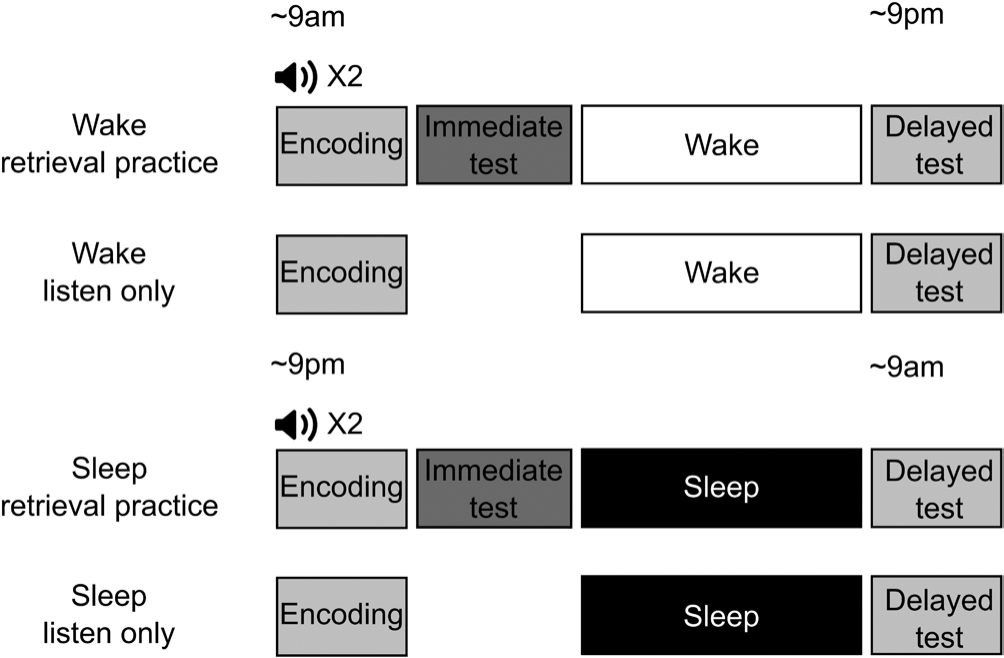
Experimental design. During encoding, participants listened twice to an audio recording of The War of the Ghosts. After a 15-minute distractor task, half of the participants were given a sheet of paper and were asked to recall the story in as much detail as possible. The other half were dismissed after listening to the story. After a 12-hour delay, all participants returned to the lab and were required to recall the story in as much detail as possible. In half of the participants, the delay included a day of wakefulness. In the other half, the delay included a night of sleep.

Participants were assigned to one of four experimental groups (Figure 1): (1) a wake + retrieval practice group (*n* = 30); (2) a wake, listen-only group (*n* = 29); (3) a sleep + retrieval practice group (*n* = 27), and (4) a sleep, listen-only group (*n* = 28). The groups did not differ in terms of age (*F*(3,110) = 0.27, *p *=* *.84, *np*^2^ =* *.007) or sex (χ^2^(3) = 0.30, *p *=* *.96), see also Supplementary Table S2. Participants listened to the War of the Ghosts story twice in succession, either in the morning (9 am), or the evening (9 pm) depending on the sleep/wake group assignment (i.e. wake groups listened in the morning, sleep groups listened in the evening). All participants then completed a 15-minute distractor task consisting of simple arithmetic problems. Next, half of the participants were dismissed (i.e. those in the two listen-only groups; wake, listen-only; sleep, listen-only) while the other half of participants were given a blank sheet of notebook paper and were instructed to engage in free recall by recollecting the story as accurately as possible (i.e. those in the two retrieval practice groups; wake + retrieval practice; sleep + retrieval practice). All participants returned 12 hours after their first session for a delayed recall test, following either a day of wakefulness (wake groups) or a night of sleep (sleep groups). For half of the participants, it was their first time recalling the story (listen-only groups), while for the other half, it was their second recall of the story (retrieval practice groups).

During each recall test, participants were encouraged to use the exact same words as heard in the story (veridical recall), and to write down as many facts and events as possible. Participants were given 10 minutes for each recall test and were encouraged to use the full-time.

### Scoring

The War of the Ghosts story was subdivided into 42 propositions following previously established methods [25, 42] (Supplementary Table S1). To facilitate identification of incorrect placements (see below), the story was demarcated into three episodes (episode 1 = lines 1–18, episode 2 = lines = 19–28, episodes 3 = lines 29–42). Handwritten transcripts of recalled material were typed into a computer and divided into units of propositions. These transcripts were then scored by two researchers who were blind to experimental condition. Interrater reliability between the two scorers showed a Cohen’s kappa of 0.84. As in previous studies using this task, all data reported below are those of scorer 1 [25]. Results were unchanged when data from scorer 2 was used. Sentences from the participant transcripts were matched to the propositions in the story that were most similar in content. We then identified propositions from the recalled material that fit into one of several categories (see Table 1 for an overview and example of each category).

**Table 1.** Rules for scoring each proposition in the story

Proposition	Rule	Example
Accurate	Propositions that were recalled verbatim, or verbatim with minor omission that did not result in a loss of detail	Verbatim: “They escaped to the shore, and hid behind some logs” With minor omission: “They escaped and hid behind some logs”
Omission	Omission of key details of the proposition	“Now canoes came up, and they heard the sound of paddles” changed to “canoes were approaching, and they started to hear noises”
Modification	Contained all elements of the proposition but used distinctly different wording, but the meaning was essentially correct	“I will not go along. I might be killed. My relatives don’t know where I have gone” changed to “But my family won’t know where I have gone and I might die”
Inference	Wrote an event in a proposition that was not explicitly stated, but could reasonably be assumed to have occurred between two events	Writing that one of the boys got into the canoe, when it is never explicitly stated that he traveled with the warriors
Normalization	Conventionalization of a story element	Recalling that the two young men went on a fishing trip, rather than going down to the river to hunt seals
Incorrect placement	The proposition contained an element from a different portion of the story	Referring to the warriors as ghosts at the beginning of the story, when they are not referred to as ghosts until the middle
Importation	An entirely new element was added to the story	Recalling that the warriors fought with guns (the story only mentions bows and arrows)

First, propositions were categorized as being either exactly correct (i.e. verbatim) or essentially correct (i.e. verbatim with minor omission that did not result in a loss of detail). These two categories were collapsed together to represent accurate memory [25]. Propositions were categorized as inaccurate if they included at least one major distortion, or if changes to the phrasing of the proposition rendered it incorrect. Inaccurate propositions were then categorized as either containing omission (a loss of detail without any distortion, i.e. detail is omitted, but nothing is added to the proposition) or containing a distortion (if the recalled proposition contains anything that modified or changes the meaning of the proposition as it is recalled). Finally, the type of distortion was identified. *Modification* was defined as a change in the phrasing of a proposition that does not add new information (see Inference) and does not change into something more common (see Normalization) but nevertheless uses a distinctly different phrasing or involves an accurate summarizing statement. *Inference* involved a change or addition to the proposition such that what was not stated before but was implied or can be inferred from the story is stated explicitly. *Normalization* was defined as changing an element in the proposition from the actual element to something more in line with that of the target social group (in this case, Harvard University undergraduates) to be the normal experience or conception of the world. An *Incorrect placement* was where a story element is imported from another episode within the story. Finally, an *Importation* occurred when an entirely new element (essentially an intrusion from an unknown source) was added to the story. The categorization of propositions was consistent with prior research [25].

### Statistical analysis

All statistical analyses were performed in *R* [43] using the *rstatix* package [44].

#### Time of day influences.

We compared immediate memory performance between the two retrieval practice groups via a series of unpaired *t*-tests, with each of the proposition types entered as a DV.

#### Baseline sleep influences.

We compared the four groups on baseline subjective sleep metrics (as obtained through sleep diaries) and alertness (as obtained through Stanford sleepiness scale assessments) via a series of 2 (delay group: sleep, wake) × 2 (study condition: retrieval practice, listen-only) ANOVAs with the following dependent variables: bedtime the night before the encoding session, waking up time the morning of the encoding session, total sleep time the night before encoding, subjective sleep quality the night before encoding, SSS score prior to encoding, and SSS score prior to the delayed test. For the two sleep groups, we also compared differences in bedtime, waking up time, total sleep time, and sleep quality for the night of sleep between encoding and delayed test between the retrieval practice and listen-only group, via a series of unpaired *t*-tests.

#### Aim 1: How does sleep and retrieval practice impact memory accuracy?

We examined the roles of sleep and retrieval practice on the number of accurate and false (i.e. importations) propositions at the delayed test by way of two separate 2 (delay group: sleep, wake) × 2 (study condition: retrieval practice, listen-only) ANOVAs. A corrected significance level of *p* value × 2 tests was used. Post hoc tests were corrected for multiple comparisons as appropriate.

#### Aim 2a: For inaccurate memories, does sleep and/or retrieval practice primarily contribute to a simple loss of episodic detail, or a distortion of the original story?

We tested how the number of omissions and distortions varied between our factors of interest. We again performed two 2 × 2 ANOVAS, with the number of omissions and the number of distortions at the delayed test entered as DVs. A corrected significance level of *p* value × 2 tests was used. Post hoc tests were corrected for multiple comparisons as appropriate.

#### Aim 2b: Which kind of memory distortions are most impacted by sleep and retrieval practice?

A series of four 2 × 2 ANOVAS were run to disentangle the effects of sleep and retrieval practice on each type of distortion made during the delayed test (modification, inference, normalization, incorrect placement). A corrected significance level of *p* value × 4 tests was used. Pairwise follow-up tests, corrected for multiple comparisons, were performed as appropriate.

We also sought to quantify the relative effects of sleep and retrieval practice on memory performance. Using the wake, listen-only group as a baseline (i.e. the group with neither sleep nor retrieval practice), we compared the size of improvements gained via either sleep (sleep, listen-only group), retrieval practice (wake, retrieval practice group), or both (sleep, retrieval practice group) by subtracting the average wake, listen-only delayed test score from each participant’s score in the other three groups [37, 45]. We then tested whether the size of improvement was significantly different from zero via a series of one-sample *t*-tests, separately for the three groups. We also directly compared the three groups (sleep, listen-only; wake, retrieval practice; sleep, retrieval practice) on their relative improvement size via ANOVA. Multiple comparison correction was applied as appropriate. To reduce the number of comparisons, we only performed these analyses for proposition categories that were significantly modulated by either delay group or test session in the ANOVA analyses described above under Aims 1 and 2.

## Results

### Ruling out time of day and baseline sleep influences

First, we checked to see whether time of day affected recall by comparing memory metrics between the sleep and the wake group who engaged in retrieval practice. There were no differences in immediate free recall performance based on delay condition for any of the metrics (all *p*s > .08, all *d*s < 0.46; Supplementary Table S3). Similarly, there was no difference between the sleep and wake groups in terms of total number of propositions produced at the initial test (*t*(54.80) = 0.96, *p *=* *.34, *d *= 0.26). At the delayed test, a 2 (delay group: sleep, wake) × 2 (study condition; retrieval practice, listen-only) ANOVA found a significant main effect of delay on total number of propositions produced (*F*(1,110) = 11.82, *p *<* *.001, *np*^2^ = .10), with more propositions in the sleep groups (*M* = 29.3, *SD* = 4.06) compared to the wake groups (*M* = 26.4, *SD* = 5.02). The main effect of study condition and the interaction were both nonsignificant (*p*s > .07).

Subjective sleep quality and alertness measures can be found in Supplementary Table S2. On the night before encoding, participants in the wake groups reported waking up significantly earlier than participants in the sleep groups (*F*(1,109) = 23.72, *p *<* *.001, *np*^2^ = .18). There was no effect of study condition (*F*(1,109) = 2.22, *p *=* *.14, *np*^2^ = .02), nor was there an interaction (*F*(1,109) = 0.42, *p *=* *.52, *np*^2^ = .004). Despite this, there were no delay group differences in subjective alertness at either session (*p*s > .096), and time of awakening did not correlate with the number of propositions made at either the immediate (*r *=* *0.10, *p *=* *.62), or delayed (*r* = −0.002, *p *=* *.98) test. No other effects of delay group, study condition, on any interactions, were significant (*p*s > .054).

### Effects of sleep and retrieval practice on delayed recall performance

Participants in the sleep groups reported sleeping on average for 424 minutes (*SD* = 56 minutes) during the night between encoding and the delayed test. There was no difference between the listen-only and retrieval practice study conditions in any of the sleep diary measures (all *p*s > .11).

**Figure 2.**
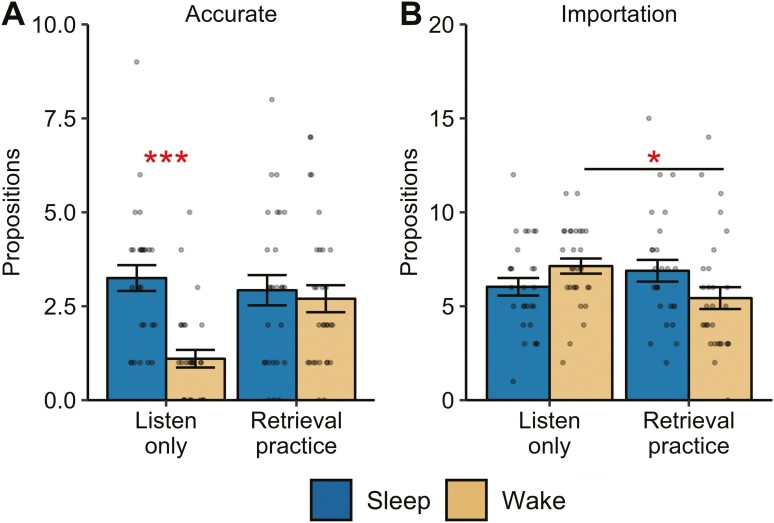
Number of propositions classified as accurate or importation (false memory) at the delayed test. Dots represent individual data points, and error bars display the between-subjects standard error of the mean. ***p <* *.001, *p <* *.05.

Number of propositions at the delayed test, as a function of category, is displayed in Supplementary Table S4. As a first analysis, we examined the effect of delay group (sleep, wake) and study condition (retrieval practice, listen-only) on memory accuracy and number of importations. For memory accuracy, we observed a main effect of delay group (*F*(1,110) = 12.23, *p*_corr_* *=* *.001, *np*^2^* *=* *.10), with more accurate propositions in the sleep groups (*M* = 3.09, *SD* = 1.95) compared to the wake groups (*M* = 1.91, *SD* = 1.83). We also observed a significant delay group × study condition interaction (*F*(1,110) = 8.02, *p*_corr_ = .012, *np*^2^*= *.068; Figure 2A). Follow-up tests revealed that accuracy was significantly higher after sleep compared to wake only for those participants who did not engage in retrieval practice (*t*(48) = 5.16, *p *<* *.001, *d* = 1.37). When participants engaged in retrieval practice, there was no difference in accuracy between the sleep and wake groups (*t*(53.5) = 0.42, *p *=* *.68, *d* = 0.11). This effect was driven by higher accuracy in the wake, retrieval practice group compared to the wake, listen-only group (*t*(49.7) = 3.72, *p* < .001, *d* = 0.97), with no differences between the two sleep conditions (sleep, retrieval practice vs sleep, listen-only) (*t*(51.5) = 0.61, *p *=* *.54, *d *= 0.17). We note that the significant interaction remained when a potential outlier was removed (*F*(1,109) = 6.88, *p*_corr_ = .020, *np*^2^ =* *.059).

For the importation of new story elements, we again observed a significant delay group × study condition interaction (*F*(1,110) = 6.21, *p*_corr_ = .028, *np*^2^ =* *.05; Figure 2B), driven by a significantly higher rate of importation of nonstory elements in the wake, listen-only group, compared to the wake, retrieval practice group (*t*(51.3) = 2.41, *p *=* *.019, *d* = 0.63). All other pairwise comparisons were nonsignificant (all *p*s > .10).

**Figure 3.**
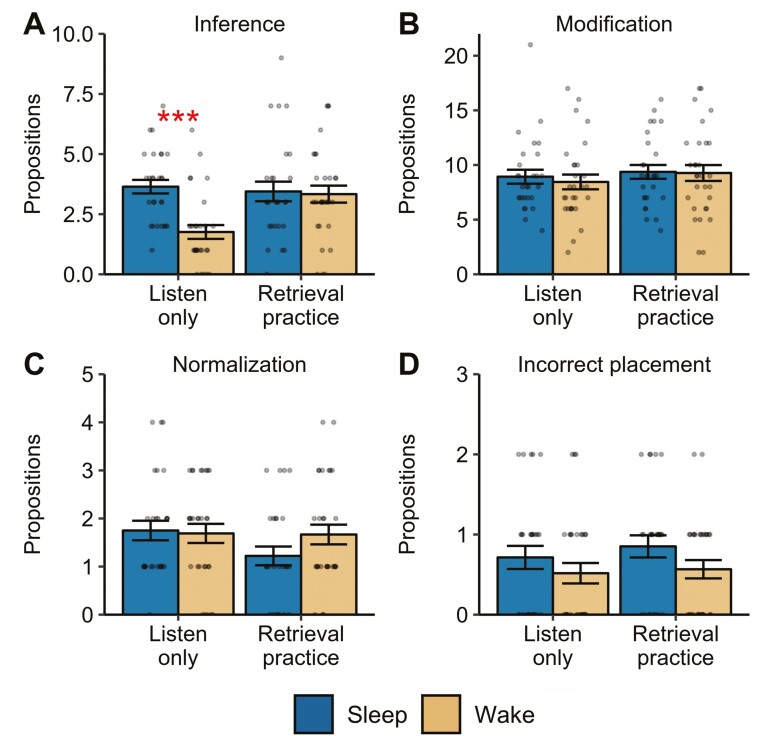
Effects of sleep and retrieval practice on distortion of the original story material. Dots represent individual data points, and error bars display the between-subjects standard error of the mean. ***p < .001.

Next, we examined the effects of sleep and testing on types of inaccurate memory (i.e. the number of omissions and distortions). There were no significant main effects or interactions between delay group and study condition on the number of omissions or distortions (all *p*s > .057). To explore potential differences within the subtypes of distortion, we examined the four different kinds of distortion separately (Figure 3). For the number of inferences made at the delayed test, there was a significant main effect of delay group (*F*(1,110) = 8.82, *p*_corr_* *=* *.016, *np*^2^* *=* *.074), with overall more inferences in participants who slept (*M* = 3.55, *SD* = 1.81) compared with participants who stayed awake (*M* = 2.56, *SD* = 1.91). This effect was superseded by a significant delay group × study condition interaction (*F*(1,110) = 6.97, *p*_corr_* *=* *.04, *np*^2^ =* *.060; Figure 3A). The number of inferences made by the sleep group was higher than the wake group when there was no retrieval practice (*t*(55) = 4.67, *p *<* *.001, *d *= 1.24), but there was no difference when retrieval practice occurred (*t*(53) = 0.21, *p *=* *.84, *d* = 0.05). The number of inferences made by the sleep group did not differ depending on whether participants engaged in retrieval practice or not (*t*(53) = 0.40, *p *=* *.69, *d* = 0.11). However, wake participants made significantly more inferences in the retrieval practice condition, compared to the listen-only condition (*t*(57) = 3.44, *p *=* *.001, *d *= 0.90). There were no main effects or interactions for modifications, normalizations, or incorrect placements made at the delayed memory test (all *p*_corr_ > .067; Figure 3B–D). No significant main effects or interactions for modifications were found after removal of an outlier (*ps* > .17).

**Figure 4.**
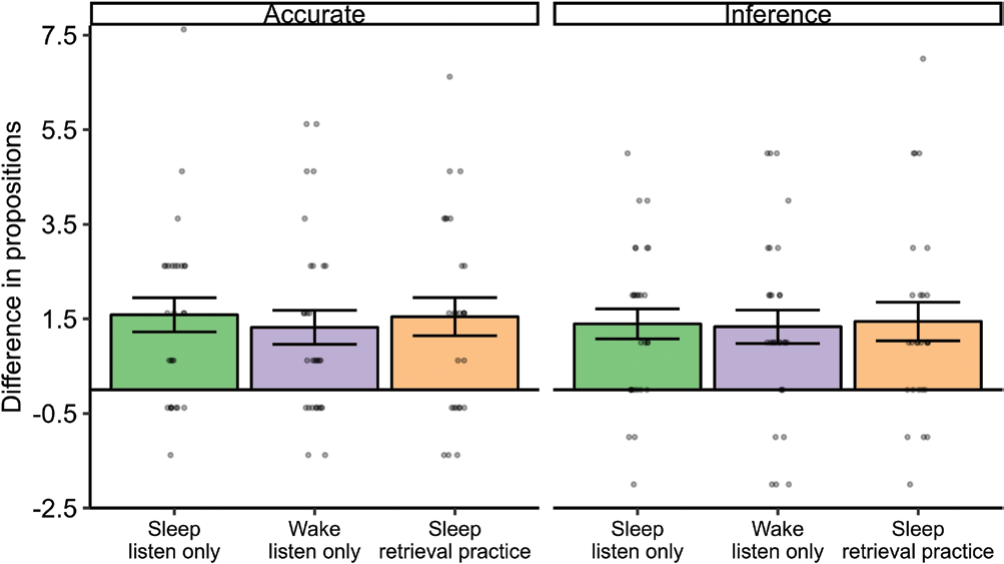
Relative effects of sleep and wake + retrieval practice on delayed memory performance. Difference scores were calculated by subtracting mean accuracy/inference from the wake, listen-only group from each participant’s score in the other three groups. As such, a positive value indicates a relative increase with sleep and/or retrieval practice, and a negative value indicates a relative decrease. Error bars show the between-subjects standard error or the mean.

These results suggest that there was an effect of sleep on increasing both accuracy and inference. However, this was true only in the absence of retrieval practice. We next sought to quantify the relative effects of sleep and retrieval practice on memory performance (Figure 4). Compared to the wake, listen-only group, both sleep and retrieval practice led to a significant improvement in accuracy, and significantly more inferences being made (all *p*s < .001, all *d*s > 0.66). Sleep and retrieval practice produced nearly identical changes in memory (accuracy increase: sleep *M* [*SD*] = 1.58 [1.91], retrieval practice *M* [*SD*] = 1.32 [1.97]; inference increase: sleep *M* [*SD*] = 1.39 [1.69], retrieval practice *M* [*SD*] = 1.33 [1.94]). The influences of sleep and retrieval practice were not additive, with the sleep, retrieval practice group not showing any significant differences compared to the sleep, listen-only or wake, retrieval practice groups (main effect of group: *F*(2,82) = 0.14, *p *=* *.87, *np*^2^ =* *.003). Aside from an overall reduction in number of propositions made, we did not observe any significant changes in propositions between the initial and delayed test in the two retrieval practice groups (all *p*s > .07).

## Discussion

Here, we studied how two key processes, namely overnight sleep and post-learning retrieval practice, impacted memory for the War of the Ghosts story over a 12-hour delay. Our key findings were that, compared to a day of wake without retrieval practice, both sleep and retrieval practice independently resulted in more accurate propositions being produced 12 hours later, but at the same time, led to significantly more inferences of plausible, but not actually presented, story elements. This finding adds further evidence that the testing effect can be as powerful as sleep for long-term memory accuracy [33], and also extends the literature to show testing also contributes to memory evolution, again in a manner similar to sleep. Although such transformations can be beneficial in terms of memory flexibility and generalization, there are also times that such distortions of the original event are unwanted (e.g. eyewitness testimony). This is also an important consideration for educators, when considering the impacts of testing and sleep on the retention and accurate recall of educational materials.

Our finding that sleep resulted in improved accuracy compared to wake fits with the large body of evidence that sleep strengthens memories for facts and events. For example, memory for word pairs is significantly better after a period of sleep compared to an equivalent period of wake [37, 45]. The present work shows that this benefit extends to a story-based task, similar to other work [46]. This finding is important, as it provides evidence that sleep’s benefit to memory translates to a more ecologically valid test of memory.

We also found that participants who slept made more inferences than participants who stayed awake. As well as being strengthened, memories undergo transformation processes during sleep. One of these transformations involves improvements in relational memory, the ability to generalize new information across existing stores of knowledge. Prior experimental work has suggested that making new inferences about the relationships between abstract visual stimuli benefits from time, and especially sleep [47, 48]. In the present study, we defined an inference as a proposition that contained an event not explicitly stated in the story but that conceivably could have occurred between two explicitly described events. The fact that these occurred more frequently after sleep may reflect the sleeping brain’s ability to generalize aspects of the story and utilize existing knowledge structures to form gist-consistent inferences regarding how events in the story likely linked together.

These effects were not unique to sleep. Post-learning retrieval practice, followed by a day of wake, produced similar changes in terms of behavior (i.e. numerically similar changes in proposition number), when compared to the wake, listen-only group (i.e. retrieval practice increased both accuracy and inference). There was no additive effect of sleep and retrieval practice, as the group that engaged in both retrieval practice and a night of sleep did not differ in delayed recall performance from the groups that received one or the other. One interpretation of this finding is that the changes in memory induced by retrieval practice altered the underlying memory representations to such a degree that sleep could not act on that representation further. This interpretation is supported in that, aside from an overall decline in total number of propositions, there were no significant changes in propositions between the immediate and delayed test in the retrieval practice groups. A growing body of work has demonstrated other instances in which presleep retrieval practice precludes any benefit of sleep, compared to a restudy condition [32–34]. Although our findings appear to support this literature, we note that in the present study, we contrasted retrieval practice to a no retrieval, listen-only condition.

Together, our results replicate and extend previous work showing that memory effects that were once ascribed to being sleep-dependent can also be achieved through retrieval practice. Thus, future work should address the similarities and differences between how retrieval practice and sleep work to benefit memory. In a recent study, Liu and Ranganath found that while retrieval practice benefitted memory for associations that occurred in close temporal proximity, sleep uniquely facilitated the retention of related information that was learned in temporally distant episodes [49]. The authors proposed that this sleep-specific effect could be due to properties of the consolidation process that are unique to sleep, whereby sleep consolidation mechanisms extend the reach of retrieval practice by allowing the brain to discover links between temporally distant experiences [49].

At a neural level, it is proposed that sleep-associated memory benefits occur through a process of active systems consolidation, whereby memory traces are repeatedly reactivated, leading to a strengthening of neocortical memory representations and altering hippocampal–cortical connectivity [50–53]. Recent work has shown that repeated testing can induce similar systems consolidation effects on the neural substrates of memory [54], leading to the suggestion that retrieval practice initiates a rapid consolidation process, resulting in similar structural and functional changes as a night of sleep [55]. As such, it is plausible that sleep and retrieval practice may impact memory via similar changes to the underlying memory, albeit on different timescales. Although we did not see any additive effects of sleep and retrieval practice, recent work has shown that long-lasting changes to memory representations achieved via retrieval practice will only be long-lasting if they are solidified by subsequent sleep [56]. An intriguing hypothesis for how sleep and retrieval practice may differ is that retrieval practice can rapidly initiate systems consolidation, but sleep is required for long-term stabilization.

Another potentially fruitful area for future research is to further examine how different types of memory might benefit differently from retrieval practice and sleep. Earlier studies have established that sleep and retrieval practice can both benefit memory for word pair associates and spatial location [32–34]. The present study extends this to memory accuracy and distortion in a more naturalistic story-based task. One explanation of these findings is that retrieval practice strengthens the underlying memory to such an extent that further consolidation during sleep is unneeded [33]. This is consistent with Dr. Stickgold’s sleep-dependent memory-triage model, which suggests that sleep consolidates new information in a discriminatory manner, targeting the most relevant memories for consolidation [26]. Regarding memory strength in particular, Dr. Stickgold has suggested that memories with a very low or high initial strength may be triaged out of subsequent sleep-related processing [10, 37]. Therefore, if retrieval practice pushes memory strength above the “sweet spot” for sleep-dependent triage, the relative benefit of sleep on these memories will be reduced.

We found that the overall number of propositions made was higher in the sleep groups compared to the wake groups, irrespective of whether participants engaged in retrieval practice or not. Thus, this effect appeared to be unique to sleep. One possible interpretation is that sleep influenced the type of strategy participants engaged in during retrieval, such that sleep participants took a more liberal approach to writing down propositions they thought they recalled. It is also possible that participants in the sleep groups were simply more motivated at the final test. Though we do note that levels of subjective alertness did not differ between the sleep and the wake groups.

A number of limitations of the work should be considered. First, as already stated, we did not contrast retrieval practice to a restudy condition as is common in the testing effect literature. We do note however that some restudy occurred in all groups, as participants listened to the War of the Ghosts story twice during encoding. Even with this limitation, our results show that reengaging with studied material can boost memory in a manner equivalent to sleep, which is important in the context of other literature showing presleep testing reduces the magnitude of the sleep effect [32–34]. Second, although our sample size was consistent with many sleep and memory studies [38–40], we did not perform an a priori power analysis due to the lack of a known effect size for this paradigm. Although the lack of an a priori power analysis is a limitation of the present work, the reported data provide an important baseline for which future high-powered studies can estimate expected effect sizes from. Third, we did not collect any data on the experiences of the participants in the wake group between encoding and the delayed test. Therefore, we don’t know whether participants rehearsed any of the material between the sessions. Fourth, we did not collect any objective measurements regarding participant’s sleep. As such, the quality and architecture of sleep following learning were unknown, and we do not know if any sleep stage of feature was specifically associated with any of the observed memory transformations. Finally, the scores in some of the proposition categories were very low, leaving open the possibility of floor effects in the case of those variables.

We found that both sleep and retrieval practice strengthened accurate recall of the story, while also inducing an increase in story-related inferences. These findings illustrate the importance of both processes in memory evolution. When considering our work in the light of Dr. Stickgold’s contributions to the field, two key themes emerge. First, our finding that inferences increase over time aligns with his suggestion that *memory evolution* is an appropriate phrase to refer to the many ways in which memories are transformed by post-encoding processes beyond simple strengthening [10]. Second, our finding that post-learning retrieval practice can promote the same memory changes as sleep can be viewed in the context of a memory-triage process [26], whereby the act of retrieval practice strengthens memory to a point that no additional reprocessing during sleep is needed [10, 33]. Future research should now further seek to determine memory processes that are unique to sleep, with a particular focus on the temporal distance between related episodes [49] and initial memory strength [10] as cues for sleep-dependent memory triage.

## Supplementary Material

zpae083_suppl_Supplementary_Tables_S1-S4

## Data Availability

The data sets generated and/or analyzed during the current study are available on the Open Science Framework, https://doi.org/10.17605/OSF.IO/3B4HG.
