# Supplementary material for: Sleep and retrieval practice both strengthen and distort story recollection
Source: Sleep Adv. 2024 Nov 16;5(1):zpae083. doi: 10.1093/sleepadvances/zpae083 (PMC11648565; doi:10.1093/sleepadvances/zpae083)
Supplement: zpae083_suppl_Supplementary_Tables_S1-S4 [file zpae083_suppl_supplementary_tables_s1-s4.pdf]

Sleep and retrieval practice both strengthen and distort story recollection

Dan Denis[1*], Carissa DiPietro[2], R. Nathan Spreng[3], Daniel L. Schacter[4], Robert Stickgold[5,6], and Jessica D. Payne[2]

[1] Department of Psychology, University of York, York, YO10 5DD, UK
[2] Department of Psychology, University of Notre Dame, Notre Dame, IN 46556, USA
[3] Montreal Neurological Institute, McGill University, Montreal, QC H3A 2B4, Canada
[4] Department of Psychology, Harvard University, Cambridge, MA 02138, USA
[5] Department of Psychiatry, Beth Israel Deaconess Medical Center, Boston, MA 02215, USA
[6] Department of Psychiatry, Harvard Medical School, Boston, MA 02215, USA

* Corresponding author:

Dan Denis
PS/B/213 Psychology Building,
University of York,
Heslington, York,
YO10 5DD,
United Kingdom

email: dan.denis@york.ac.uk

Supplementary materials

Table S1. The War of the Ghosts

1	One night two young men from Egulac went down to the river to hunt seals,
2	and while they were there it became foggy and calm.
3	Then they heard war cries,
4	and they thought, “Maybe this is a war party”
5	They escaped to the shore
6	and hid behind some logs.
7	Now the canoes came up,
8	and they heard the noise of paddles
9	and saw one canoe coming up to them.
10	There were five men in the canoe,
11	and they said, “What do you think? We wish to take you along.
12	We are going up the river to make war on the people.”
13	One of the young men said, “I have no arrows”
14	“Arrows are in the canoe” they said.
15	“I will not go along.
16	I might be killed.
17	My relatives do not know where I have gone.
18	But you,” he said, turning to the other, “may go with them”
19	So one of the young men went,
20	But the other returned home.
21	And the warriors went on up the river to a town on the other side of Kalama.
22	The people came down to the water,
23	and they began to fight,
24	and many were killed.
25	But presently the young man heard one of the warriors say, “Quick, let us go home; that Indian has been hit.”
26	Now he thought, “Oh, they are ghosts”
27	He did not feel sick,
28	but they said he had been shot.
29	So the canoes went back to Egulac,
30	and the young man went ashore to his house and made a fire.
31	And he told everybody and said, “Behold, I accompanied the ghosts, and we went to a fight
32	Many of our fellows were killed,
33	and many of those who attacked us were killed.
34	And they said I was hit
35	and I did not feel sick.”
36	He told it all,
37	And then he became quiet.
38	When the sun rose he fell down.
39	Something black came out of his mouth.
40	His face became contorted.
41	The people jumped up and cried.
42	He was dead.

Table S2. Demographics, subjective sleep, and alertness measures

	Wake, listen only M (SD)	Sleep, listen only M (SD)	Wake + retrieval practice M (SD)	Sleep + retrieval practice M (SD)
Age (years)	20.1 (1.7)	19.9 (1.7)	20.4 (2.3)	20.1 (2.11)
Sex				
Female	45%	64%	57%	52%
Male	55%	36%	43%	48%
Pre-study sleep log[1]				
Bedtime	00:49 (01:26)	01:12 (01:15)	00:29 (00:56)	01:05 (01:35)
Wake time	08:01 (00:45)	09:04 (01:05)	07:51 (00:46)	08:39 (01:23)
Total sleep time (mins)	431 (74)	472 (59)	442 (61)	454 (97)
Sleep quality[2]	2.24 (0.62)	2.32 (0.55)	2.40 (0.62)	2.50 (0.58)
Consolidation night sleep log				
Bedtime	-	01:30 (00:58)	-	01:05 (00:59)
Wake time	-	08:23 (00:35)	-	08:22 (00:41)
Total sleep time (mins)	-	414 (52)	-	437 (59)
Sleep quality[2]	-	2.36 (0.56)	-	2.31 (0.55)
Session 1 subjective alertness[3]	2.83 (1.00)	2.46 (0.74)	2.83 (0.83)	2.65 (0.90)
Session 2 subjective alertness	2.54 (1.14)	2.55 (1.18)	2.57 (1.14)	2.85 (1.01)

Note. Sex expressed as a percentage of the group. All other variables show M = Mean, SD = Standard deviation. [1] Pre-study sleep log measures reflect the average of the three nights prior to the start of the experiment. [2] Sleep quality rated on a 1-3 scale, where 1 = poor, 2 = average, 3 = good. [3] Subjective alertness assessed by the Stanford sleepiness scale. A larger number indicates feeling more sleepy/less alert (theoretical range 1-7).

Table S3. Number or propositions exhibiting each memory category at the immediate memory test

	Wake + retrieval practice M (SD)	Sleep + retrieval practice M (SD)	*p*
Accurate	3.27 (2.41)	3.04 (2.33)	.716
Importation	4.20 (2.34)	5.15 (2.66)	.161
Omission	5.23 (1.98)	5.33 (2.32)	.862
Distortion (total)	14.6 (3.93)	15.1 (2.73)	.623
Modification	10.3 (3.74)	10.4 (3.07)	.935
Inference	2.60 (1.87)	2.56 (1.85)	.928
Normalization	1.33 (1.15)	1.44 (1.40)	.746
Incorrect placement	0.37 (0.67)	0.67 (0.68)	.100

Note. M = mean, SD = standard deviation. *p* values (uncorrected) reflect unpaired t-tests used to compare the two groups.

Table S4. Number of propositions exhibiting each memory category at the delayed recall test

	Wake, listen only M (SD)	Sleep, listen only M (SD)	Wake + retrieval practice M (SD)	Sleep + retrieval practice M (SD)	*p*
Accurate	1.10 (1.26)	3.25 (1.82)	2.70 (1.97)	2.93 (2.09)	**.006**
Importation	7.14 (2.17)	6.04 (2.47)	5.43 (3.18)	6.89 (3.00)	**.014**
Omission	4.55 (1.84)	4.61 (1.71)	4.53 (1.91)	4.93 (1.94)	.628
Distortion (total)	12.40 (4.24)	15.0 (3.56)	14.80 (4.17)	14.90 (2.62)	.069
Modification	8.45 (3.63)	8.93 (3.38)	9.27 (3.98)	9.37 (3.28)	.780
Inference	1.76 (1.55)	3.64 (1.50)	3.33 (1.94)	3.44 (2.12)	**.010**
Normalization	1.69 (1.07)	1.75 (1.08)	1.67 (1.12)	1.22 (1.01)	.212
Incorrect placement	0.52 (0.69)	0.71 (0.76)	0.57 (0.63)	0.85 (0.72)	.737

Note. M = Mean, SD = standard deviation. p-values (uncorrected) reflect the interaction between delay group (Sleep, Wake) and study condition (Retrieval practice, Listen only)
